# Relationship Between Perivascular Fat Inflammation and Coronary Atherosclerotic Plaque Composition

**DOI:** 10.3390/jcm15041652

**Published:** 2026-02-22

**Authors:** Leif-Christopher Engel, Rafael Adolf, Salvatore Cassese, Erion Xhepa, Adnan Kastrati, Michael Joner, Heribert Schunkert, Martin Hadamitzky, Philipp Nicol

**Affiliations:** 1 Department of Cardiovascular Diseases, German Heart Center, TUM University Hospital, 80636 Munich, Germany; casesse@dhm.mhn.de (S.C.); xhepa@dhm.mhn.de (E.X.); kastrati@dhm.mhn.de (A.K.);; 2 Department of Cardiovascular Radiology and Nuclear Medicine, German Heart Center Munich, TUM University Hospital, 80636 Munich, Germany; rafadolf@hotmail.com (R.A.);

**Keywords:** fat attenuation index, coronar computed tomography angiography, plaque composition, perivascular inflammation

## Abstract

**Background:** Perivascular fat attenuation index (FAI) derived from coronary CT angiography (CCTA) has emerged as a quantitative biomarker of vascular inflammation, with potential to improve risk stratification in coronary artery disease (CAD) patients. This study aimed to evaluate plaque characteristics of coronary atherosclerotic lesions in patients with high (≥−70.1 HU) or low FAI of pericoronary adipose tissue. **Methods:** In a retrospective analysis, patients with suspected or confirmed CAD who underwent coronary CTA were included. Coronary lesions were classified into two groups based on their perivascular inflammation as assessed by CCTA: high perivascular FAI phenotype (≥−70.1 HU) versus low FAI phenotype (<−70.1 HU). Both groups were compared with respect to various patient- and lesion-specific characteristics. **Results:** A total of 247 coronary lesions were analyzed in this study. Of these, 36 (14.6%) lesions were associated with high perivascular inflammation (high FAI phenotpye) and 211 (85.4%) were associated with low perivascular inflammation (low FAI phenotype). Lesions with a high FAI phenotype demonstrated a significantly higher amount of non-calcified plaque volume (NCPV) compared to lesions with a low FAI phenotype [(111.8 mm^3^ (69.4–184.2) versus 87.7 mm^3^ (44.6–143.0), *p* < 0.003]. NCPV emerged as a consistent and significant predictor of fat attenuation positive plaque in both univariate (OR 1.030 [95% CI, 1.010–1.050], *p* = 0.003); and multivariate logistic regression analyses (OR 1.028 [95% CI, 1.008–1.050]. *p* = 0.007). Additionally, lesions with a high FAI phenotype less frequently exhibited homogeneous calcification than their low FAI phenotype counterparts (25% versus 46.9%, *p* = 0.014). **Conclusions**: Coronary lesions associated with a high FAI phenotype on coronary CCTA consist predominantly of non-calcified plaques. Conversely, lesions characterized by a low perivascular FAI phenotype are primarily calcified and seem to be more homogeneous by visual assessment. Further prospective studies are warranted to validate these associations and explore the underlying pathophysiological mechanisms.

## 1. Introduction

Identification and characterization of atherosclerotic lesions is crucial for effective management and interventional strategies in coronary artery disease (CAD) patients. Recent advancements in imaging modalities, particularly coronary computed tomography angiography (CCTA), have enhanced our ability to visualize coronary anatomy and pathology [1,2,3,4]. The perivascular fat attenuation index (FAI), derived from CCTA, has emerged as a potential biomarker for assessing the inflammatory state of atherosclerotic plaques. The perivascular fat attenuation index offers a quantitative assessment of coronary inflammation, which may enhance the prediction of cardiac risk beyond existing evaluation methods [5,6,7,8]. Especially highly elevated FAI values of ≥−70.1 HU of pericoronary adipose tissue (PCAT) serve as significant indicators of increased cardiac mortality and can inform tailored prevention strategies for this patient group [5]. CCTA is a powerful tool for analyzing not only the surrounding FAI but also for accurately assessing plaque composition, including high-risk plaque characteristics that are strongly linked to major adverse cardiovascular events, such as acute coronary syndrome and sudden cardiac death [9,10]. Currently, more research is necessary to precisely define the specific plaque features associated with very high pericoronary FAI values. This study aimed to evaluate the relationship between PCAT of extremely high perivascular FAI ≥ −70.1 HU and plaque characteristics of coronary atherosclerotic lesions in patients with symptomatic CAD.

## 2. Materials and Methods

### 2.1. Study Population

This study analyzed retrospective data from the German Heart Center Munich, specifically targeting patients with suspected or confirmed CAD. The dataset comprises individuals who underwent both coronary CT angiography (CCTA) and invasive catheterization during the period of 2011–2018. We selectively included individuals with symptoms suggestive of CAD who required invasive angiography and percutaneous coronary intervention due to the presence of obstructive CAD on CCTA. Although used as a surrogate for baseline risk, the diagnostic data obtained during invasive angiography were not included as variables in the final analysis. Patients with acute coronary syndrome, hemodynamic instability, unstable sinus rhythm during the examination, prior stent placement, or previous coronary bypass surgery were excluded from the analysis. Before the examination, a structured assessment was conducted to gather data on age, height, weight, cardiac history, current concerns, and medications. In these patients, coronary atherosclerotic lesions were classified into two groups based on their perivascular inflammation as assessed by CCTA: lesion with a high perivascular FAI phenotype (≥−70.1 HU) versus lesion with low FAI phenotype (<−70.1 HU). Both groups were compared with respect to various patient and lesion-specific characteristics. The study protocol was reviewed and approved by the local institutional ethics committee.

### 2.2. Image Acquisition

CCTA acquisition was performed using successive generations of dual-source CT technology (Siemens Healthineers, Erlangen, Germany). From January 2011 to May 2014, imaging was conducted on a 128-slice system, which was subsequently superseded by a 192-slice dual-source platform for the remainder of the study period through December 2018. To optimize image quality, intravenous beta-blockers were administered based on patients’ heart rates and the absence of contraindications, aiming to achieve a heart rate of less than 60 beats per minute. If the systolic blood pressure exceeded 100 mmHg, sublingual nitrates were administered. Coronary prospective ECG-synchronized CTA was gated to the diastolic phase, specifically at 70% of the R-R interval, utilizing a high-pitch, single-acquisition protocol. While tube voltage was tailored at the discretion of the clinician (ranging from 70 to 120 kVp), the tube current was dynamically modulated to the patient’s habitus using automated dose exposure control (CARE Dose). Contrast circulation time was established using a test bolus of 10 mL of contrast media (Imeron 350, Bracco Imaging GmbH, Konstanz, Germany), followed by a 50 mL saline chaser. The coronary CT angiogram was executed with a 50 mL contrast bolus administered at a rate of 5.0 mL/s, succeeded by a 30 mL saline administration. Axial images were reconstructed during the cardiac end-diastolic phase, employing a slice thickness of 0.6 mm with a 0.4 mm reconstruction increment to ensure optimal spatial resolution.

### 2.3. Assessment of CCTA Data

A specialized semi-automatic software prototype (Coronary Plaque Analysis 2.0.3, Syngo.via VA30, Siemens, Erlangen, Germany) was employed to analyze the morphological characteristics of coronary plaques. Following automated centerline extraction and vessel labeling, inner and outer vessel walls were delineated using CPR. To ensure accuracy, cross-sectional views were manually optimized to remove side branches and venous interference. Two blinded observers (R.A. and L.-C.E.) independently assessed all lesions; disagreements were resolved through consensus. Diameter and area stenosis were calculated by referencing the average dimensions of the unaffected vessel segments flanking the lesion.

Lesion locations were mapped to the 15-segment AHA model. Plaque characterization was performed via automated volumetric analysis based on tissue-specific attenuation: lipid-rich components were defined by a range of −100 to 70 HU, fibrotic tissue by 71 to 124 HU, and calcified plaque by values exceeding 125 HU. Total and sub-component plaque volumes were subsequently calculated for each lesion.

Coronary arteries were classified as diffusely diseased if the coronary vessel wall displayed a continuous burden of atherosclerotic plaque without any non-diseased segments. Plaque morphology was qualitatively categorized as ‘eccentric’ if the calcified component occupied less than 50% of the vessel circumference. Conversely, calcification encompassing more than half of the arterial perimeter was classified as concentric. Calcified portions were classified as “spotty” if their size was less than three mm in diameter and as homogeneous when the attenuation within the calcified part remained consistent. Furthermore, the following features were considered high-risk plaque characteristics: “spotty” calcification, positive remodeling (as indicated by a remodeling index >1), a non-calcified plaque volume exceeding 60%, and stenosis greater than 70%. A coronary artery plaque was classified as predominantly calcified or non-calcified if the corresponding content of calcified or non-calcified plaque exceeded 60%.

Pericoronary adipose tissue (PCAT) attenuation was quantified radially from the external vessel wall within a volume of interest defined by the vessel’s internal diameter. Adipose tissue was isolated using a voxel-specific attenuation range of −190 to −30 HU, as previously validated. We further stratified these values to identify lesions associated with an elevated fat attenuation index (FAI), defined by an attenuation range of −30 to −70 HU. This shift in perivascular adipose tissue (PVAT) composition—characterized by a transition from lipid-rich (closer to −190 HU) to more aqueous, less fatty phenotypes (closer to −30 HU)—serves as a robust surrogate for vascular inflammation and a prognostic indicator of major adverse cardiac events (MACE).

### 2.4. Statistics

Statistical analyses were performed using IBM SPSS Statistics, version 24 (IBM Corp., Armonk, NY, USA). Continuous variables are presented as means ± standard deviation (SD) or as medians (interquartile range) for non-normally distributed data. Comparisons between groups were conducted using the unpaired Student’s *t*-test or the Mann–Whitney U test, as appropriate. Categorical variables were evaluated via the chi-square test. To identify predictors of a high-FAI phenotype, univariate and multivariate logistic regression analyses were employed. Multivariate models were refined using backward elimination, sequentially removing variables with the highest *p*-values until only those with significant *p*-values remained. Statistical significance was defined as two-tailed.

## 3. Results

A total of 247 coronary lesions found in 184 patients were analyzed in this study. Patients were stratified into two groups based on the FAI of pericoronary adipose tissue surrounding a coronary lesion: patients with at least one lesion with a high pericoronary FAI phenotype (values ≥ −70.1 HU) (n = 24) and patients with a low pericoronary FAI phenotype (values < −70.1 HU) (n = 160). Clinical baseline characteristics and medical treatment on admission are presented in Table 1. Across the two groups, there were no significant differences, with the exception of the levels of triglycerides. Of the 247 coronary lesions, 36 (14.6%) were associated with high perivascular inflammation (fat attenuation positive) and 211 (85.4%) were associated with low perivascular inflammation (fat attenuation negative).

In subjects with lesions with a high FAI phenotype, the most lesions were located in the right coronary artery (RCA; n = 19, 52.8%), followed by the left anterior descending artery (LAD; n = 10, 27.8%), and the left circumflex artery (LCX; n = 7, 19.4%). In subjects with lesions with a low FAI phenotype, the majority of lesions were located in the left anterior descending artery (LAD; n = 125, 59.2%) followed by the right coronary artery (RCA; n = 52, 24.6%), the left circumflex artery (LCX; n = 28, 13.3%) and the left main stem (n = 6, 2.8%).

Lesions associated with a high and low FAI phenotype demonstrated no differences with respect to the degree of stenosis (77.3 ± 15.8% versus 73.9 ± 18.5%; *p* = 0.290), minimal lumen area.

[MLA; 1.64 (0.77–2.41) versus 1.71 (0.91–3.05), *p* = 0.258)], lesion length (28.2 ± 10.3 versus 27.5 ± 8.8 mm, *p* = 0.673), and remodeling index (1.02 ± 0.41 versus 1.05 ± 0.51, *p* = 0.784). Furthermore, both lesions associated with high and low perivascular inflammation were similarly likely to be associated with diffusely diseased vessels (47% versus 36.5%; *p* = 0.176). Likewise, differences in total plaque volumes between both groups were not statistically significant [187.4 mm^3^ (136.4–332.3) versus 197.3 mm^3^ (126.6–316.9), *p* = 0.779] and both groups did not differ with respect to the presence of eccentric appearance (67% versus 70.1%, *p* = 0.675).

In terms of plaque composition, lesion with a high FAI phenotype demonstrated a significantly higher amount of non-calcified plaque volume compared to fat attenuation negative lesions [(111.8 mm^3^ (69.4–184.2) versus 87.7 mm^3^ (44.6–143.0), *p* < 0.003]. In contrast, lesions with a low FAI phenotype consisted of significantly higher amount of calcified plaque volume [101.8 mm^3^ (61.5–173.6) versus 80.2 mm^3^ (66.9–104.3), *p* = 0.003]. Univariate logistic regression suggested a strong association between calcified plaque volume and lesions associated with a low FAI phenotype (OR 0.973 [95% CI, 0.954–0.991], *p* = 0.003); however, this relationship did not remain statistically significant in the multivariate model (OR 1.023 [95% CI, 0.873–1.199], *p* = 0.729). In contrast, non-calcified plaque volume emerged as a consistent and significant predictor of lesions with a high FAI phenotype in both univariate (OR 1.030 [95% CI, 1.010–1.050], *p* = 0.003) and multivariate logistic regression analyses (OR 1.028 [95% CI, 1.008–1.050]. *p* = 0.007). Univariate and multivariate logistic regression of CCTA characteristics for lesions with a high FAI phenotype are seen in Table 2.

**Figure 1:**
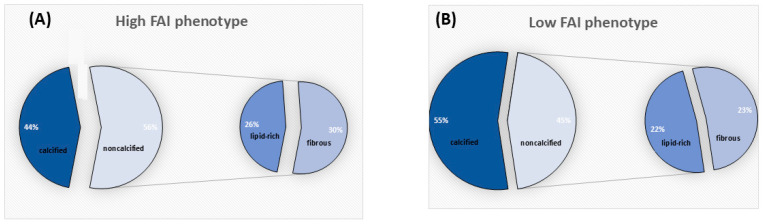
Distribution of Plaque Components Based on Perivascular Inflammation: (**A**) Comparison of plaque components between lesions with high perivascular inflammation (i.e., high FAI phenotype) and (**B**) low perivascular inflammation (i.e., low FAI phenotype) revealed a clear compositional difference. Lesions with a high FAI phenotype exhibited a higher proportion of non-calcified material and a lower proportion of calcified material. Conversely, lesions with a low FAI phenotype were characterized by a predominance of calcified components. Analyzing the non-calcified portions further, the amount of the fibrous component was observed to be only slightly greater than the lipid-rich component in lesions with a high FAI phenotype. In contrast, these non-calcified components were almost equally distributed in lesions with a low FAI phenotype (e.g., fibrous 23% versus lipid-rich 22%).

**Figure 2:**
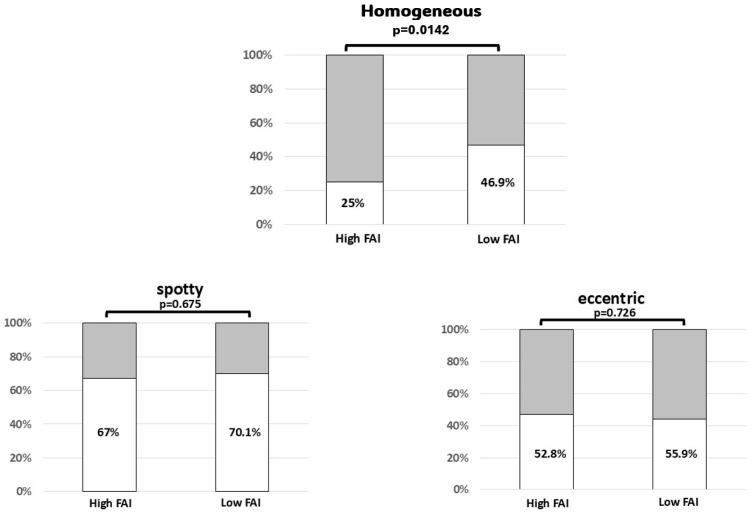
The characteristics of calcified plaque segments were compared between lesions associated with a high and a low FAI phenotype: Calcification within plaques associated with low perivascular inflammation (low FAI phenotype tended toward greater homogeneity than that observed in lesions with high perivascular inflammation (i.e., high FAI phenotype). However, the two groups showed no significant difference regarding the presence of spotty or eccentric calcification (grey part of the bar represents the absence of a homogeneous, spotty or eccentric plaque characteristic).

Coronary atherosclerotic plaques marked by high fat attenuation index contained a greater quantity of non-calcified plaque and less calcification (56% and 44%, respectively). Conversely, lesions with a low FAI phenotype carried predominantly calcified components (55% calcified versus 45% non-calcified). Within the non-calcified segment, fibrous material (30%) was marginally more prevalent than lipid-rich material (26%) in lesions with a high FAI phenotype. However, in lesions with a low FAI phenotype, these non-calcified components were nearly balanced (e.g., fibrous 23% vs. lipid-rich 22%) (see Figure 1). When looking specifically at the calcification in both groups, the data demonstrated that lesions with a high FAI phenotype less frequently exhibited homogeneous calcification than their low FAI counterparts (25% versus 46.9%, *p* = 0.014). Both groups did not differ with respect to spotty calcification (52.8% versus 55.9%, *p* = 0.726) (see Figure 2).

## 4. Discussion

High fat attenuation index (FAI) values greater than −70.1 HU of pericoronary adipose tissue (i.e., high FAI phenotype lesions) are established as a crucial, noninvasive surrogate marker for vascular inflammation, demonstrating clear links to adverse cardiac outcomes and cardiac mortality [5,6,7]. Our findings from CCTA analysis demonstrate a significant association between increased perivascular fat attenuation and the composition of atherosclerotic lesions in patients with CAD.

Specifically, coronary atherosclerotic lesions characterized by a high perivascular FAI greater than −70.1 (i.e., high FAI phenotype lesions) predominantly contained non-calcified plaque components, suggesting a more unstable and inflammatory phenotype. In contrast, lesions with low perivascular FAI < −70.1 (i.e., low FAI phenotype lesions) were primarily composed of calcified plaques, indicating a more stable atherosclerotic profile. This distinction is critical, as non-calcified plaques are often associated with a higher risk of acute coronary events [2,3].

The PCAT itself is a metabolically active endocrine organ, vital for regulating cardiovascular homeostasis through its complex balance of inflammatory and anti-inflammatory components [11,12,13,14,15,16,17]. In essence, PCAT does more than just store energy; it is actively involved in cardiac metabolism and significantly influences the adjacent coronary artery. Changes in PCAT can release inflammatory adipokines, which accelerate plaque development and make existing plaques more vulnerable [11,12,13]. Previous methodological studies have validated the reliability of PCAT proximal to the right coronary artery (RCA) for inflammation detection due to its advantageous anatomical properties [5,6,7]. Crucially, the present study deviates from prior work by measuring FAI values of PCAT specific to each lesion, based on the compelling hypothesis that inflammation is most pronounced in the immediate perilesional environment.

**Figure 3:**
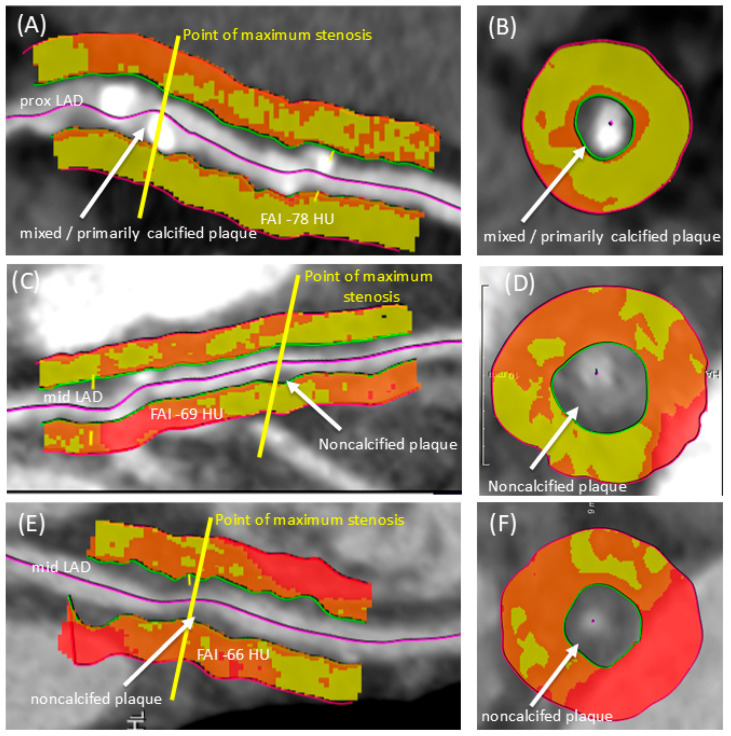
Representative images illustrate the morphological distinctions in coronary atherosclerotic lesions based on perivascular inflammation, categorized by fat attenuation (high versus low FAI phenotype). At the point of maximum stenosis, visualized in both long- and short-axis views, lesions exhibiting low perivascular inflammation (<−70.1 HU) tend to be characterized by a predominantly calcified plaque composition (**A**,**B**), whereas lesions associated with high perivascular inflammation (≥−70.1 HU) display a greater content of non-calcified plaque burden (**C**,**D**). Completely non-calcified lesion as seen in (**E**,**F**) were never part of the high FAI phenotype group.

Existing literature has firmly established that elevated PCAT attenuation values correlate directly with increased local coronary inflammation causing a poorer clinical outcome [5,7]. While some studies have shown correlations between PCAT markers and non-calcified plaques that vanish after covariate correction, others have documented a higher lesion-specific fat attenuation index for non-calcified plaques and mixed plaques compared to calcified plaques [18]. Our findings substantiate and extend this body of work: We observed a greater amount of non-calcified plaque volume at the expense of calcified plaque volume in lesions with extremely high FAI values (see Figure 3). This phenomenon is plausibly explained by the release of inflammatory factors from non-calcified plaque portions, which induces adjacent perivascular wall edema and inhibits local adipogenesis, thereby raising the FAI of the pericoronary adipose tissue [14,15,16,17].

While we found a strong correlation between the volume of soft plaque (non-calcified plaque) and the degree of inflammation around the vessel (perivascular inflammation), the anticipated connection with spotty calcification, which is a marker for vulnerable, unstable plaques and poor patient outcomes, was absent. Spotty calcification was evenly distributed in lesions, regardless of whether they had high or low perivascular inflammation. This surprising result is likely due to our study cohort consisting of high-risk patients with existing stenotic coronary artery disease. Consequently, a selection bias was introduced, meaning that even the group with less perivascular inflammation already represented a population with significant vascular compromise beyond the norm.

However, a specific comparison of calcified plaque segments revealed a notable correlation between perivascular inflammation and calcification homogeneity. To the best of our knowledge, this is the first study to characterize in detail the calcified components of plaque in direct relation to perivascular inflammation, filling a significant gap in the current literature. In particular, our data demonstrated that lesions associated with high perivascular inflammation (i.e., high FA phenotype lesions) less frequently exhibited homogeneous calcification than their low FAI phenotype counterparts. Homogeneous calcification on CCTA has been interpreted as a hallmark of stability and appears as a smooth, uniform area of high density within the plaque, indicating the calcium deposits are well-integrated and have a consistent internal structure. This finding, the uniform, dense appearance of the plaque, is therefore regarded as a low-risk feature, indicating that the lesion is less likely to be associated with adverse cardiac outcomes than more unstable morphologies like spotty calcification or mixed plaques [19,20].

Several limitations must be considered. Among others, the study is a retrospective study with no clinical outcome data or hard clinical endpoints. Additionally, the group with lesions associated with a high FAI phenotype had a relatively small sample size and this study lacks a comparison with other inflammatory imaging methods, such as positron emission tomography. Consequently, the extent of plaque inflammation may not be fully validated in this study. Finally, the inclusion of a high-risk cohort with prior invasive angiography and PCI introduce a significant selection bias. This is a major limitation with respect to the generalizability of the findings to lower-risk or screening populations.

## 5. Conclusions

Our study indicates that coronary lesions with a high FAI phenotype on CCTA carry a higher non-calcified plaque volume. Conversely, lesions characterized by a low FAI phenotype are primarily calcified and seem to be more homogeneous by visual assessment. These findings may underscore the potential of perivascular FAI as a crucial imaging biomarker for identifying high-risk atherosclerotic lesions in patients with CAD, further prospective studies are warranted to validate these associations and explore the underlying pathophysiological mechanisms.

## Figures and Tables

**Table 1:** Baseline patients’ characteristics and medical treatment on admission.

Characteristics	Patients with High FAI Phenotype Lesions (n = 24)	Patients with Low FAI Phenotype Lesions (n = 160)	*p*-Value
Age, y	62.4 ± 7.9	65.5 ± 8.5	0.09
Male, n (%)	17 (47.2)	127 (60.2)	0.145
BMI, kg/m^2^	25.9 ± 2.7	26.7 ± 2.6	0.148
**Risk factors**			
Hypercholesterolemia, n (%)	11 (45.8)	87 (54.4)	0.434
Hypertension, n (%)	16 (66.7)	104 (65.0)	0.872
Smoking, n (%)	9 (37.5)	48 (30.0)	0.458
Family history of CAD, n (%)	9 (37.5)	38 (23.8)	0.149
Diabetes melitus, (%)	12 (50.0)	98 (61.3)	0.294
**Laboratory findings**			
Creatinine, mg/dL	0.84 ± 0.19	0.89 ± 0.14	0.245
C-reactive protein, mg/dL	3.8 ± 5.02	3.0 ± 2.9	0.396
Total cholesterol, mg/dL	202.8 ± 32.5	198.9 ± 37.2	0.358
Triglyceride, mg/dL	188.0 ± 116.5	140.2 ± 64.2	**0.021**
HDL cholesterol, mg/dL	52.8 ± 13.3	56.9 ± 14.7	0.238
LDL cholesterol, mg/dL	119.1 ± 24.2	118.8 ± 33.8	0.489
Hemoglobin A1c, %	5.7 ± 0.5	5.9 ± 0.6	0.201
**Medication**			
Aspirin	6 (25)	52 (32.5)	0.460
Statin	5 (20.8)	60 (37.5)	0.111
Beta-blocker	6 (25.0)	52 (32.5)	0.460
ACEI and/or ARB	6 (25.0)	61 (38.1)	0.213
Insulin, n (%)	0 (0)	2 (1.3)	0.368
OAD, n (%)	0 (0)	9 (5.6)	0.656

Abbreviations: BMI, body mass index; CK, creatinine kinase; ACEI, angiotensin-converting enzyme inhibitor; ARB, angiotensin II receptor blocker; OAD, oral antidiabetic drugs.

**Table 2:** Univariate and multivariate logistic regression of CCTA characteristics for fat attenuation positive culprit lesions.

CCTA Variable	High FAI Phenotype n = 36	Low FAI Phenotype n = 211	Univariate Analysis		Multivariate Analysis	
			OR (95% CI)	*p*-Value	OR (95% CI)	*p*-Value
Stenosis, %	77.3 ± 15.8	73.9 ± 18.5	1.011 (0.990–1.033)	0.290	0.999 (0.974–1.025)	0.939
MLA	1.64 (0.77–2.41)	1.71 (0.91–3.05)	0.888 (0.723–1.091)	0.258	0.960 (0.784–1.175)	0.692
Lesion length, mm	28.2 ± 10.3	27.5 ± 8.8	1.008 (0.970–1.049)	0.673	0.997 (0.939–1.057)	0.911
RI	1.02 ± 0.41	1.05 ± 0.51	0.896 (0.407–1.970)	0.784	0.777 (0.342–1.768)	0.547
Diffusely diseased, n (%)	17 (47)	77 (36.5)	1.644 (0.800–3.376)	0.176	1.436 (0.674–3.059)	0.349
Total PV (mm^2^)	187.4 (136.4–332.3)	197.3 (126.6–316.9)	1.000 (0.998–1.002)	0.779	0.999 (0.997–1.001)	0.462
Calcified PV (mm^2^)	80.2 (66.9–104.3)	101.8 (61.5–173.6)	0.972 (0.954–0.991)	0.003	1.023 (0.873–1.199)	0.779
Non-calcified PV (mm^2^)	111.8 (69.4–184.2)	87.7 (44.6–143.0)	1.030 (1.010–1.050)	0.003	1.028 (1.008–1.050)	0.007
Spotty calcification n (%)	17 (47)	93 (44.1)	1.135 (0.559–2.306)	0.726	0.876 (0.382–2.008)	0.755
Eccentric calcification n (%)	24 (66,7)	148 (70.1)	0.851 (0.401–1.808)	0.675	1.351 (0.556–3.283)	0.507
Multiple lesions, n (%)	11 (30.6)	39 (18.5)	1.941 (0.881–4.275)	0.100	1.491 (0.601–3.404)	0.418

Abbreviations: MLA, minimal lumen area; RI, remodeling index; PV, plaque volume.

## Data Availability

The datasets generated and/or analyzed during the current study are not publicly available due to ethical restrictions but are available from the corresponding author upon reasonable request.
